# Using demographics toward efficient data classification in citizen science: a Bayesian approach

**DOI:** 10.7717/peerj-cs.239

**Published:** 2019-11-25

**Authors:** Pietro De Lellis, Shinnosuke Nakayama, Maurizio Porfiri

**Affiliations:** 1 Department of Electrical Engineering and Information Technology, University of Naples Federico II, Naples, Italy; 2 Department of Mechanical and Aerospace Engineering, New York University Tandon School of Engineering, Brooklyn, NY, USA; 3 Department of Biomedical Engineering, New York University Tandon School of Engineering, Brooklyn, NY, USA

**Keywords:** Citizen science, Bayesian estimation, Data classification, Algorithms

## Abstract

Public participation in scientific activities, often called citizen science, offers a possibility to collect and analyze an unprecedentedly large amount of data. However, diversity of volunteers poses a challenge to obtain accurate information when these data are aggregated. To overcome this problem, we propose a classification algorithm using Bayesian inference that harnesses diversity of volunteers to improve data accuracy. In the algorithm, each volunteer is grouped into a distinct class based on a survey regarding either their level of education or motivation to citizen science. We obtained the behavior of each class through a training set, which was then used as a prior information to estimate performance of new volunteers. By applying this approach to an existing citizen science dataset to classify images into categories, we demonstrate improvement in data accuracy, compared to the traditional majority voting. Our algorithm offers a simple, yet powerful, way to improve data accuracy under limited effort of volunteers by predicting the behavior of a class of individuals, rather than attempting at a granular description of each of them.

## Introduction

Involvement of crowds in the creation of goods and services has become a powerful and successful model to achieve goals (Howe, 2006). Crowdsourcing can take various forms, which can be classified based on types of contributions and motivations, with openness to the public as a common feature (Franzoni & Sauermann, 2014; Sauermann & Franzoni, 2015). For example, some crowdsourcing platforms recruit crowdworkers to undertake microtasks (Difallah et al., 2015), and others seek for innovative ideas and solutions (Penin & Burger-Helmchen, 2012; Estellés-Arolas & González-Ladrón-de Guevara, 2012; Majchrzak & Malhotra, 2013; Cappa, Rosso & Hayes, 2019) or money (Lehner, 2013; Belleflamme, Lambert & Schwienbacher, 2014), by extrinsically motivating the crowds with rewards. Over the past decades, participation in scientific activities by public volunteers, often called citizen science, has emerged as a new tool to conduct science at an unprecedentedly large scale (Silvertown, 2009; Bonney et al., 2014). Citizen science is uniquely positioned in crowdsourcing typologies, as the crowds contribute to science through intrinsic motivation on voluntarism, rather than extrinsic motivation based on receiving rewards (Ryan & Deci, 2000; Nov, Arazy & Anderson, 2014; Cappa et al., 2018).

With prevalence of the Internet, citizen science now attracts diverse people to contribute to research projects by collecting and analyzing raw data online at their convenience. Popular and successful citizen science projects include *eBird*, where volunteers upload the locations of observed birds (https://ebird.org), and *EyeWire*, where volunteers reconstruct retinal neurons in 3D from 2D images (https://eyewire.org). Although citizen science enables scientists to acquire a large amount of processed data, it may come at the expense of data quality. Since the data are collected and analyzed by the untrained public, they might suffer from low quality, challenging contribution to science (Dickinson, Zuckerberg & Bonter, 2010; Kosmala et al., 2016; Kallimanis, Panitsa & Dimopoulos, 2017). Therefore, it is of interest to citizen science practitioners to enhance the quality of data, while making good use of volunteers’ effort.

A common practice in citizen science builds upon the wisdom of the crowd, whereby scientists distribute the same tasks to multiple participants and then aggregate the data (Swanson et al., 2015). Beyond aggregation rules, sophisticated methods have been proposed in the field of crowdsourcing to tackle the so-called noisy labeler problem (Sheng, Provost & Ipeirotis, 2008; Frenay & Verleysen, 2014). One of the most notable methods employs an expectation-maximization algorithm (Dawid & Skene, 1979), where the ground truth and the reliability of labelers are simultaneously estimated through an iterative procedure to maximize the likelihood of the model parameters. The method can also be extended into a Bayesian framework for more accurate estimation of ground truth and labeler reliability (Raykar et al., 2010; Kim & Ghahramani, 2012). However, having a granular characterization of each participant could be practically unfeasible or not convenient. Indeed, this would require every volunteer to participate in a preliminary session in which their accuracy would be thoroughly characterized. This might represent an unacceptable misuse of the volunteers’ time, and it will likely be unfeasible in realistic cases where the volunteers contribute only for a very limited time (Nov, Arazy & Anderson, 2011).

An economical solution to mitigate the redundancy of volunteers’ effort is to collect labels on the same instance repeatedly from different labelers until it meets a threshold defined by a requester (Chen, Lin & Zhou, 2013; Li et al., 2016). Further, in dynamic task allocation, a next instance to be labeled is selected from a pool of instances through a Bayesian Markov decision process, which identifies the instance that would maximize a reward function if it were labeled next (Chen, Lin & Zhou, 2013). In this way, requesters can minimize the effort of labelers, while maintaining adequate data quality. However, the basic algorithm assumes that all labelers have equal reliability, which is unlikely true in citizen science. While the approach can be extended to estimate both ground truth and labeler reliability simultaneously in sequential task allocation, it might become unfeasible in citizen science to accurately estimate reliability of each volunteer with only a few instances of labels (Nov, Arazy & Anderson, 2011).

Thus far, the diversity of volunteers in citizen science poses a challenge to accurately estimating the ground truth, but it may be possible to turn the tables and harness this diversity to enhance data accuracy. Since citizen science welcomes everyone by nature, volunteers belong to a wide demographic, with diverse age and educational level (Cappa et al., 2016; Burgess et al., 2017), as well as motivations (Nov, Arazy & Anderson, 2014; Curtis, 2015; Cappa et al., 2018). These individual attributes could provide additional information toward enhancing data accuracy while safeguarding volunteers’ effort. For example, the motivational level explains both quality and quantity in citizen science (Nov, Arazy & Anderson, 2014), and the educational level is positively related to the accuracy of identifying invasive species (Delaney et al., 2008). In a Bayesian sense, this information may help enhance data accuracy by affording an informative prior distribution of reliability for each individual attribute.

A Bayesian framework has been used by Garriga, Piera & Bartumeus (2017) to evaluate and rank participants in citizen science projects based on their reputation, with the final goal of increasing the likelihood of engagement and the overall data quality. Here, we investigate the possibility of employing a Bayesian approach to enhance classification accuracy by harnessing diversity of volunteers in citizen science. Specifically, this study aims at improving the accuracy of noisy data by incorporating information about demographics of volunteers into a Bayesian framework and dynamically distributing tasks among a limited number of volunteers. We use data collected within a citizen science project, the Brooklyn Atlantis (Laut et al., 2014), where volunteers performed binary classification tasks. The study aimed at monitoring the environment of the Gowanus Canal (Brooklyn, NY), a highly polluted body of water in the USA. Volunteers were presented with images of the Canal and asked to classify the objects in the images, by assessing whether they might represent a threat to the environment (Torre et al., 2019). Before classifying the image, they were asked selected demographic information, which were not analyzed in Torre et al. (2019), whose focus was on improving data accuracy by providing a possibility to cast blank votes in a classification task. Specifically, the degree of interest of the volunteers toward the environment and their level of education were recorded.

Using the dataset of Torre et al. (2019), we applied a Bayesian approach that leverages these individual attributes for enhancing the classification efficiency. To validate the approach, we allocated volunteers randomly to tasks until the theoretical accuracy of the classification overcomes a chosen threshold. We computed the average classification accuracy and number of volunteers employed as performance metrics, and compared them against the traditional majority voting approach.

## Methods

### Data collection

The data used in this study were collected within a citizen science project for obtaining information about the status of the environmental health of the Gowanus Canal (Brooklyn, NY, USA) (Torre et al., 2019). The images were taken by an aquatic robot designed as part of the Brooklyn Atlantis project (Laut et al., 2014), which, over the years, was used to address a number of important questions in citizen science, from the effect of design interventions to face-to-face interactions with scientists and on to improving engagement in rehabilitation exercises (Laut et al., 2015, 2017; Nov, Laut & Porfiri, 2015; Cappa et al., 2016, 2018; Palermo et al., 2017a, 2017b; Diner et al., 2018; Nakayama et al., 2018; Torre et al., 2019).

Volunteers were asked to inspect the images of the Canal and identify the presence of objects that could endanger the environment (Torre et al., 2019). The images taken by the robot were uploaded on a temporary website built for this experiment, where volunteers could access them from their computers and mobile devices. Before taking part in the project, participants had to log in through either a Facebook profile or an email account to prevent them from performing the task more than once. After accessing the website, participants were first presented with a short movie about the scope of the project. Then, participants initiated a practice session, in which they were instructed to classify whether the object in the image would represent a potential threat to the environment by clicking either a “threat,” “no threat,” or “I don’t know” button below the image. After the task was performed, the correct answer was shown together with a brief explanation. Before the experiment, Torre et al. (2019) identified the correct answer of each image through careful examination and discussion, and the selection of images only included those which received a unanimous classification.

After the classification of two objects in the practice session, the main task started, and participants were asked to classify 31 images consecutively, which appeared on the screen for 5 s each. Participant could choose between “threat,” “no threat,” or “I don’t know” buttons, but this time, the correct answer was not displayed. If the participant did not select any answer in 5 s, it was recorded as “no answer.” To avoid possible confounding effects on performance, the order of the images’ display was randomized for each participant. Upon completing the classification task, the participants were asked to fill out a short questionnaire where they provided information on their education level and degree of interest toward the environment.

The data collection was carried out between February and June 2017, with a total of 91 volunteers recruited in the project. Here, we focus on the 88 of them who filled out the preliminary demographic questionnaire. All the participants were over 18 years old and their responses were anonymized. The data collection was approved by the institutional review board of New York University (IRB-FY2016-184).

### Bayesian inference

Let us assume that a pool ${\mathcal V} = \left\{ {1, \ldots ,n} \right\}$ of volunteers participates in the binary classification of a set ${\mathcal I} = \left\{ {1, \ldots ,m} \right\}$ of images. In the process of classification of image $i \in {\mathcal I}$, the unobservable binary parameter that we wish to estimate is denoted as θ_*i*_. In our experiment, θ_*i*_ is equal to 1 if image *i* contains a threat for the environment, and it is equal to 2 otherwise. A priori, we assume that we have no cues on the possible content of that image, and therefore we set

$${P_0}\left({{{\rm \theta}_i} = 1} \right) = {P_0}\left({{{\rm \theta} _i} = 2} \right) = 0.5$$

for all *i*, where the subscript 0 indicates that we refer to the probability at step 0, that is, before starting the classification process. After every successive classification, we propose to sequentially update these probabilities by using Bayes’ rule (Gelman et al., 2013). At each classification step, say *j* ≥ 1, the observable data is the classification *y_il_* of image *i* performed by participant *l* = *l*(*j*), randomly selected from the pool ${\mathcal V}$ at step *j*. The possible outcomes of the observed variable *y_il_* are 0, corresponding to a late reply (the participant does not classify within 5 s), 1 or 2, corresponding to the participant classifying the image as containing or not containing a threat, respectively, and 3, corresponding to an uncertain participant choosing the “I don’t know” option.

In a Bayesian framework, the behavior of the *l*-th participant is characterized by the conditional probabilities

(1) $$P({y_{il}} = {\rm \alpha} |{{\rm \theta} _i} = \rm \beta),$$

for all α ∈ {0,1,2,3}, β ∈ {1,2}, and $i \in {\mathcal I}$. Since we do not know a priori whether some images are more difficult to classify than others, we assume that the probabilities in (1) are independent of *i*, and therefore, for all $i \in {\mathcal I}$, we write

(2) $$P({y_{il}} = {\rm \alpha} |{{\rm \theta}_i} = {\rm \beta}) = P({y_l} = {\rm \alpha} |{{\rm \theta}_i} = {\rm \beta}),$$

which represents the probability that the classification output of the *l*-th participant is equal to α, given that the image contains (or does not contain) a threat (depending on the value of β).

In this work, we propose that the behavior of a volunteer, say the *l*-th, is related to his/her demographics (such as motivations and educational level), encoded by a vector *x_l_* of one or more integer variables. More specifically, we assume that the probabilities (1) depend on the variables *x_l_*, which are therefore called *explanatory* in the Bayesian literature (Carlin, Louis & Carlin, 2000; Gelman et al., 2013; Garriga, Piera & Bartumeus, 2017). Accordingly, based on the classification performed by the participant *l*(*j*) randomly selected at step *j*, and on his/her demographics, the probability that image *i* contains a threat for the environment can be updated in a Bayesian fashion as follows:

(3) $$\matrix{{{P_j}({{\rm{\theta }}_i} = 1)} \hfill & { = {{P({y_l} = {\rm{\alpha }},{{\rm{\theta }}_i} = 1,{x_l})} \over {P({y_l} = {\rm{\alpha }},{x_l})}} = {{P({y_l} = {\rm{\alpha }}|{{\rm{\theta }}_i} = 1,{x_l}),P({x_l},{{\rm{\theta }}_i} = 1)} \over {P({y_l} = {\rm{\alpha }}|{x_l})P({x_l})}}} \hfill  \cr {} \hfill & { = {{P({y_l} = {\rm{\alpha }}\left| {{{\rm{\theta }}_i} = 1,{x_l})P({x_l}} \right|{{\rm{\theta }}_i} = 1)P({{\rm{\theta }}_i} = 1)} \over {P({y_l} = {\rm{\alpha }}|{x_l})P({x_l})}},} \hfill  \cr } $$

for all *j ≥* 1, where *P_j_*(θ_*i*_ = 1) is defined as *P_j_*(θ_*i*_ = 1|*y_l_* = α, *x_l_*), and we omit the explicit dependence of *l* on *j* to simplify the notation. Observing that *x_l_* and θ_*i*_ are independent, we have *P*(*x_l_*|θ_*i*_ = 1) = *P*(*x_l_*), thus yielding

(4) $${P_j}({{\rm \theta}_i} = 1) = {{P({y_l} = {\rm \alpha} |{{\rm \theta}_i} = 1,{x_l})P({{\rm \theta}_i} = 1)} \over {P({y_l} = {\rm \alpha} |{x_l})}}.$$

From the law of total probability, we can write

(5) $$P({y_l} = {\rm \alpha} |{x_l}) = P({y_l} = {\rm \alpha} |{{{\rm \theta}_i} = 1},{x_l})P({{\rm \theta}_i} = 1|{x_l}) + P({y_l} = {\rm \alpha} |{{{\rm \theta}_i} = 2},{x_l})P({{\rm \theta}_i} = 2|{x_l}).$$

Noting again the independence between *x_l_* and θ_*i*_, and substituting (5) into (4), we finally establish

(6) $${P_j}({\rm \theta _i} = 1) = {{P({y_l} = {\rm \alpha} |{\rm \theta _i} = 1,{x_l}){P_{j-1}}({\rm \theta _i} = 1)} \over {P({y_l} = {\rm \alpha} |{\rm \theta _i} = 1,{x_l}){P_{j-1}}({\rm \theta _i} = 1) + P({y_l} = {\rm \alpha} |{\rm \theta _i} = 2,{x_l}){P_{j-1}}({\rm \theta _i} = 2)}},$$

where we used as prior *P_j_*_− 1_(θ_*i*_ = 1).^1^ 1.This selection of the prior implies that ${P_j}\left({{{\rm \theta} _i} = 1} \right)$ is also conditioned on the classifications and demographics of participants $l(1), \ldots, l(j-1)$. Once the conditional probabilities *P*(*y_l_* = α|θ_*i*_ = β, *x_l_*), for all α, β, and *x_j_*, have been estimated on a sample of volunteers, then, as a new volunteer *v* decides to participate in the study, we only need access to the demographics *x_v_* to characterize his/her behavior.

Setting a threshold 0.5 *≤* σ <1, we label the image as classified at the first step *t ≥* 1 such that either *P_t_* (θ_*i*_ = 1) > σ or *P_t_* (θ_*i*_ = 1) < 1 − σ, and the final classification is

(7) $$\hat{{\hskip3pt}{\rm \theta} _i} = \arg \mathop {\max }\limits_{\rm \beta \in \{ 1,2\} } {P_t}({{\rm \theta} _i} = {\rm \beta}).$$

The threshold σ can be viewed as the selected confidence level for the classification. Clearly, the higher σ is, the higher the accuracy would be, but this would require a larger number of volunteers to classify the image.

The effectiveness of the Bayesian inference is intrinsically related to our knowledge of the conditional probabilities in Eq. (6). If these probabilities were fully known, the more explanatory variables we considered, the faster *P_j_*(θ_*i*_ = 1) would converge to either 0 or 1, thereby leading to a more efficient classification for a given confidence level σ. However, in real applications we can only perform sample estimations of these conditional probabilities, which are typically evaluated on a small dataset. Therefore, their accuracy might be undermined by the sample size, but also by a biased demographic distribution of the sample. Hence, a trade-off arises in the choice of the explanatory variables: adding variables increases the theoretical classification accuracy, but the sample estimation might become less accurate due to the reduced size of the sample on which the conditional probabilities are estimated. Therefore, in designing a Bayesian classification algorithm, a crucial point is the selection of how many and which explanatory variables should be considered.

### Classification algorithm

We consider the degree of interest toward the environment and the level of education of the volunteers as possible explanatory variables. The interest toward the environment is encoded by the integer *x_l_*_1_, ranging from 1 (participant *l* is “not at all” interested) to 5 (participant *l* is “very much” interested), while the education level is encoded by a second integer parameter *x_l_*_2_, which increases from 1 (“high school diploma or less”) to 4 (“graduate or professional degree”) as the participant education level increases, while it is set to 5 if he/she prefers not to answer. Accordingly, this yields three possible choices for *x_l_*: the behavior of the participant can be evaluated based only on the degree of interest toward the environment (*x_l_* = *x_l_*_1_), on the education level (*x_l_* = *x_l_*_2_), or on both explanatory variables (*x_l_* = [*x_l_*_1_ *x_l_*_2_]^*T*^, where the superscript *T* means matrix transposition).

For any possible choice of *x_l_*, adopting a Bayesian approach for classification requires a preliminary estimation of the participants’ accuracy based on their demographics. Specifically, this consists in estimating the conditional probabilities

(8) $$P({y_l} = {\rm \alpha} |{\rm \theta _i} = {\rm \beta}, {x_l}),$$

for all α ∈ {0, 1, 2, 3}, β ∈ {1, 2}, and all possible values of *x_l_*. To this aim, we consider the set of volunteers ${\mathcal V}$ who filled out the demographic questionnaire, and partition it in two groups, denoted ${\mathcal T}$ and ${\mathcal C}$, respectively. The set ${\mathcal T}$ encompasses the volunteers used to compute the sample estimations

(9) $$\widehat P({y_l} = {\rm \alpha} |{\rm \theta _i} = {\rm \beta}, {x_l})$$

of the conditional probabilities in Eq. (8), and is called *training set* in the following, while the set ${\mathcal C} = {\mathcal V} - {\mathcal T}$ is used for testing the performance of the Bayesian approach. Namely, each image $i \in {\mathcal I}$ is classified as a result of the following steps:

- *Initialization*: the prior is set to ${\widehat P_0}({\theta _i} = \beta) = {P_0}({\theta _i} = \beta) = 0.5$, β = 1, 2, and the set of volunteers available for classification at step 0 is ${{\mathcal A}_0} = {\mathcal C}$; a threshold σ is selected in the interval $\left({0.5,1} \right)$;
- *Step j ≥* 1: a participant *l* = *l*(*j*) is randomly selected in ${{\mathcal A}_{j - 1}}$, which is updated as
  (10) $${{\mathcal A}_j} = {{\mathcal A}_{j - 1}} - \left\{ {l(j)} \right\},$$
  and the estimated probabilities ${\widehat P_j}(\rm \theta = {\rm\beta})$, β ∈ {1,2}, leveraging the sample estimations (9), are computed as
  (11) $$\hskip-7pt{\widehat P_j}({{\rm \theta} _i} = 1) = {{\widehat P({y_l} = {{\rm \alpha}_{il}}|{{\rm \theta}_i} = 1,{x_l}){{\widehat P}_{j-1}}({{\rm \theta}_i} = 1)} \over {\widehat P({y_l} = {{\rm \alpha} _{il}}|{{\rm \theta}_i} = 1,{x_l}){{\widehat P}_{j-1}}({{\rm \theta}_i} = 1) + \widehat P({y_l} = {{\rm \alpha}_{il}}|{{\rm \theta}_i} = 2,{x_l}){{\widehat P}_{j-1}}({{\rm \theta}_i} = 2)}},$$
  and ${\widehat P_j}({\theta _i} = 2) = 1-{\widehat P_j}({\theta _i} = 1)$, where α_*il*_ is the output of the classification of image *i* performed by participant *l*; and
- *Termination*: the algorithm terminates at the first step *t* such that either ${{\mathcal A}_t} = \emptyset $ or
  (12) $$\max \left\{ {{{\widehat P}_t}({{\rm \theta} _i} = 1),{{\widehat P}_t}({{\rm \theta}_i} = 2)} \right\} \gt {\rm \sigma} .$$
  Similar to Eq. (7), the *i*-th image is classified as
  ${\hat \theta _i} = \arg \mathop {\max }\limits_{{\rm \beta} \in \{ 1,2\} } {\widehat P_t}({{\rm \theta}_i} = {\rm \beta}),$
  and the number of participants used to classify image *i* is recorded as *n_i_* = *t*.

### Performance analysis

Out of the 91 volunteers who participated in the study, we focus on the 88 who filled out the questionnaire, so that $\left| {\mathcal V} \right| = 88$. Our goal is to determine whether the Bayesian approach can successfully leverage demographic information, and which individual attributes should be used as a proxy of reliability. Furthermore, we seek to evaluate the impact on the overall performance of the termination threshold σ, which is varied in the set (0.5,1) with step 0.02. Then, for each value of σ and for all the three possible selections of *x_l_*, we evaluate the performance of the classification algorithm in terms of the average number *ν* of volunteers employed, computed as $\nu  = \sum_{i \in {\mathcal I}} {{n_i}} /\left| {\mathcal I} \right|,$ with *n_i_* being the number of volunteers considered to classify the *i*-th image, and of classification accuracy χ, evaluated as the fraction of the 31 images that is correctly classified. The ground truth used to evaluate χ is represented by the preliminary classification performed by Torre et al. (2019).

We notice that the performance of the classification algorithm might be biased by the choice of the set of volunteers ${\mathcal T}$ employed for estimating the conditional probabilities (8), and by the specific classification order of the volunteers in the set ${\mathcal C}$. We set the cardinality of the set ${\mathcal T}$ to 45, which is approximately half of the total number of volunteers, and, to avoid potential biases, we randomly pick *m* = 10,000 alternative selections ${{\mathcal T}_i}$, *i* = 1,…,*m*, of the set ${\mathcal T}$, and for each *i* we consider *p* = 100 random permutations of ${{\mathcal C}_i} = {\mathcal V} - {{\mathcal T}_i}$. Then, for each possible choice of σ and *x_l_*, we compute the mean values $\bar \chi $ and $\bar \nu $ as

(13) $$\bar{\rm \chi} ({\rm \sigma}, {x_l}) = {1 \over {mp}}\sum\limits_{i = 1}^m \sum\limits_{j = 1}^p {{\rm \chi} _{ij}}({\rm \sigma}, {x_l}),\quad \quad {\overline \nu}({\rm \sigma}, {x_l}) = {1 \over {mp}}\sum\limits_{i = 1}^m \sum\limits_{j = 1}^p {\nu _{ij}}({\rm \sigma}, {x_l}),$$

where χ_*ij*_ and ν*_ij_* are the accuracy and average number of volunteers employed when using ${{\mathcal T}_i}$ as the training set and considering the *j*-th permutation of ${{\mathcal C}_i}$ as the classification sequence, respectively.

For comparison purposes, we use the majority voting approach (Kestler et al., 2011) as a reference. Namely, we consider the outcome of the classification when using the same sequence and number of participants used for Bayesian estimation, and compute its average value ${\bar {\rm \chi} _{mv}}({\rm \sigma}, {x_l})$ for all σ and for all the three possible choices of *x_l_*. A complementary metric is the percentage π(σ, *x_l_*) of all trials where the accuracy of the Bayesian approach overcomes that of majority voting.

To further delve into the performance difference between the two approaches and clarify the impact of the threshold σ, we present receiver operating characteristic (ROC) curves, typically employed to compare and select binary classifiers (Fawcett, 2006). For each value of the threshold σ, the ROC curve depicts the true positive rate (TPR) against the false positive rate (FPR). The TPR is defined as the fraction of real positives (the image contains a threat) that are correctly classified as positive, while the FPR is the fraction of real negative (the image does not contains a threat) that are incorrectly classified as positive. Then, for each value of the threshold σ, we extract a scalar unbiased measure of accuracy, the area under the curve (AUC) (Powers, 2011). We remark that, as the threshold σ modulates the number of participants employed to classify an image, and not the rate of positives, the ROC curves might not be monotone as in standard ROC analysis (Fawcett, 2006).

## Results

### Preliminary analysis of the citizen science data

In total, 88 volunteers filled out the demographic questionnaire. Table 1 presents the demographic composition of the pool of volunteers, while Tables 2 and 3 describe the distribution of the classifications outputs depending on the degree of interest toward the environment and on the education level, respectively. The χ^2^ test for independence revealed that the distributions of answers were different among *x_l_*_1_ (${\rm \chi} _{20}^2$ = 100,320, *p* < 0.001) and among *x_l_*_2_ (${\rm \chi} _{10}^2$ = 25,813, *p* = 0.004). From visual inspection, we cannot identify any trivial relationship between the classification output and demographics. Lack of correlation is also supported by the Kendall rank correlation coefficients ρ_1_ and ρ_2_ between the fraction of images correctly classified and the variables *x_l_*_1_ and *x_l_*_2_, respectively. Although one might expect volunteers’ accuracy to be positively correlated both with their interest toward the environment and education, we found ρ_1_ = − 0.06 and ρ_2_ = − 0.02, suggesting an absence of a linear dependence.

**Table 1:** Demographic composition of the pool of volunteers.

*x_l_*_2_	*x_l_*_1_						
	1	2	3	4	5	N/A	Total
2	1	0	2	0	1	0	4
3	2	0	3	4	2	0	11
4	13	2	26	26	6	1	74
N/A	0	0	0	0	0	2	2
Total	16	2	31	30	9	3	91

**Note:**

N/A corresponds to non-valid answers.

**Table 2:** Counts of late responses, true positives, false positives, true negatives, false negatives, and “I don’t know” based on the interest toward the environment.

*x_l_*_1_	True positives	False positives	True negatives	False negatives	Late responses	I don’t know
1	85	118	110	124	24	35
2	8	16	14	18	1	5
3	92	244	178	261	56	130
4	103	256	205	236	41	89
5	77	63	56	42	12	29

**Table 3:** Counts of late responses, true positives, false positives, true negatives, false negatives, and “I don’t know” based on the education level.

*x_l_*_2_	True positives	False positives	True negatives	False negatives	Late responses	I don’t know
2	25	26	13	33	8	19
3	29	86	79	90	23	34
4	311	585	471	558	103	235

**Note:**

None of the participants has “high school diploma or less” (*x_l_*_2_ = 1) or preferred not to answer (*x_l_*_2_ = 5).

A closer look at the conditional distributions can help identify some non-trivial relationships between the classification outputs and demographics. For instance, from Table 2 we observe that the number of late replies is the highest when participants are “very much” interested in the environment. This could suggest that the participants are afraid to misjudge the image and then click the wrong button, due to their genuine concern for the environment. At the same time, their percentage of false positives is the lowest, so they seldom generate a false alarm. Our Bayesian estimation algorithm has the potential of leveraging this kind of less trivial nonlinear relationships between volunteers’ accuracy and demographics.

### Bayesian inference against majority voting

**Figure 1:**
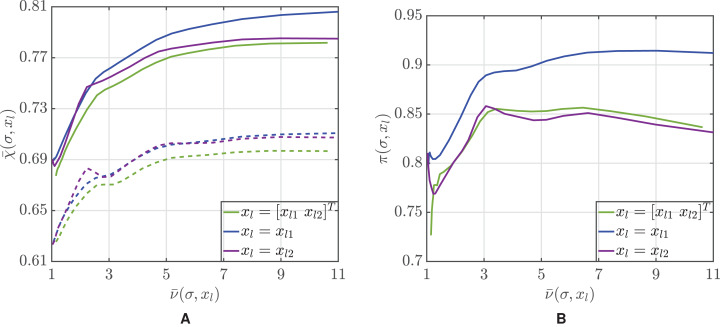
Mean accuracy $\bar {\rm \chi} $ of the Bayesian classification approach (solid lines) and of the majority voting using the same sequence of volunteers (dotted lines) (A) and percentage of trials where the Bayesian approach outperforms majority voting (B) as a function of the mean average number of volunteers $\bar {\rm \nu} $ used for classification.

In Bayesian estimation, the selection of the most appropriate explanatory variables is crucial for boosting its performance. Although in principle the more explanatory variables we include, the better estimation we attain, the finiteness of the training sample requires a more thoughtful approach. In Fig. 1, we compare the performance for the three alternative choices of *x_l_*, that is, the explanatory variables are either both the degree of interest toward the environment and education level (*x_l_* = [*x_l_*_1_ *x_l_*_2_]^*T*^), or just one of the two attributes (*x_l_* = *x_l_*_1_ or *x_l_* = *x_l_*_2_). From panel A, we see that, for all values of the threshold σ, the accuracy decreases when both explanatory variables are considered. This outcome can be explained by considering that the sample is too small ($\left| {\mathcal T} \right|$ = 45) to allow for an accurate estimation of the conditional probabilities in Eq. (1) for all the 15 possible combinations of *x_l_*_1_ and *x_l_*_2_. Furthermore, we observe that the best performance is obtained when the interest toward the environment is used as the explanatory variable. This can be expounded by looking at the demographic composition of the pool. Indeed, from Table 1 we observe a more uniform distribution of the pool with respect to *x_l_*_1_, while the level of education is skewed toward *x_l_*_2_ = 4, as more than the 81% of the participants has a graduate or professional degree. This clearly limits the accuracy in the estimation of the conditional probabilities in (1) when *x_l_*_2_ ≠ 4, thus explaining the superior accuracy associated to the choice *x_l_* = *x_l_*_1_.

The effectiveness of a Bayesian approach is also confirmed by a direct comparison with the majority voting. As one can note from Fig. 1A, for all possible choices of the explanatory variables *x_l_* and the threshold σ, the average accuracy of the Bayesian algorithm is superior to majority voting when the same sequence of labelers is used. Furthermore, in all cases the percentage *π*(σ, *x_l_*) of trials in which the Bayesian classification outperforms majority voting is larger than 72.7%, see Fig. 1B. Choosing *x_l_* = *x_l_*_1_ results in a higher performance, with a peak of *π*(σ, *x_l_*) = 92.6% when σ = 0.98.

**Figure 2:**
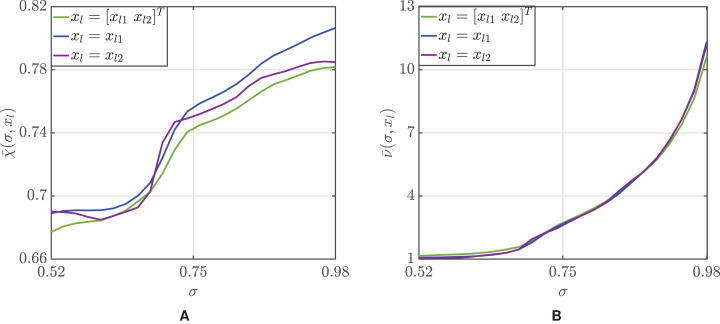
Mean accuracy (A) and mean number of participants (B) as a function of the threshold σ.

Figure 2 illustrates how the threshold σ can be used to modulate the tradeoff between accuracy and average number of volunteers employed. If the conditional probabilities (1) were known, both the accuracy and number of volunteers should monotonically increase with σ. However, this becomes nontrival when those probabilities are estimated, whereby a correct choice of the explanatory variable is crucial. For *x_l_* = *x_l_*_2_ and low values of σ the average accuracy $\bar {\rm \chi} $ decreases with σ, but, when the optimal choice *x_l_* = *x_l_*_1_ is made, monotonicity is regained and the more participants we use, the more the accuracy improves.

**Figure 3:**
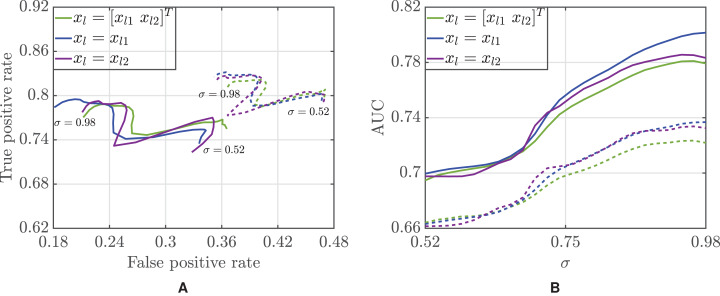
ROC curve (A) and area under the curve (B) as a function of the threshold σ for the Bayesian (solid lines) and majority voting (dotted lines) classifiers.

These considerations are further confirmed by the ROC analyses in Fig. 3, as an alternative accuracy measure, the AUC, is also non-monotone with σ for *x_l_* = *x_l_*_2_, while for *x_l_* = *x_l_*_1_ we can tune σ to regulate the tradeoff between the AUC and $\bar \nu $. Moreover, the ROC curves highlight differences between the two classifiers, where we observe a shift of the curves toward the left, such that our Bayesian classifier strongly reduces the FPR. This comes at the price of a moderate decrease of the TPR.

## Discussion

In this study, we proposed a Bayesian approach to enhance data quality in citizen science projects where sequential tasks have to be processed by a limited number of volunteers. By harnessing the diversity of participants in citizen science, we developed an algorithm that characterizes the behavior and accuracy of each participant based on his/her demographics. To demonstrate the effectiveness of our approach, we used data collected within the Brooklyn Atlantis project (Torre et al., 2019), where participants were asked to determine if selected pictures of the Gowanus Canal contained potential threats for the environment or not. Specifically, we posited that participants could be grouped in classes depending on their motivation to participate to the study, measured by their declared interest toward the environment, and on their level of education. Following a Bayesian rationale, we characterized the behavior of each class of participants on a training dataset, by estimating the probability of each possible classification output conditioned to the actual content of the image.

Our numerical analyses showed that, without resorting to a granular characterization of each participant, a Bayesian algorithm has superior performance compared with the traditional majority voting approach (Kestler et al., 2011). We were able to leverage the highly nonlinear relationships between the participants’ accuracy and their demographics toward higher accuracy, without increasing their workload. Differently from powerful alternatives to majority voting, such as the expectation maximization algorithm (Dempster, Laird & Rubin, 1977; Dawid & Skene, 1979), our approach does not require estimating the accuracy of each participant. This feature is crucial for citizen science applications, where the contribution of the volunteers might be limited to a few instances (Nov, Arazy & Anderson, 2011). In our algorithm, when a new volunteer decides to participate in the study and performs a task, his/her accuracy is immediately inferred based on demographics.

A key aspect of our Bayesian approach is the selection of the individual attributes to group participants into classes. In this study, we examined the level of education and motivation based on the literature (Nov, Arazy & Anderson, 2014; Delaney et al., 2008), but other selections are also feasible. For example, underpinned by the person-environment fit theory (Caplan, 1987), previous studies in crowdsourcing demonstrate improvement in data accuracy by matching task types with individual skills (Ho, Jabbari & Vaughan, 2013), inherent cognitive abilities (Goncalves et al., 2017), or past performance (Jung & Joon, 2014). In contrast to these studies, the advantage of our Bayesian approach lies in predicting performance of classes of individual attributes. Consequently, it can accommodate nonlinearity in the relationship between individual attributes and their performance, thereby affording more relaxed assumptions in their relationship.

Our Bayesian approach begets enhanced data accuracy with limited effort of participants by applying a prior distribution to new participants based on their demographics. This is especially advantageous in citizen science projects that involve ongoing data collection, because practitioners do not need to recalibrate the prior distribution. However, it is necessary to do so when the nature of some new tasks or the demographics of the new participants is substantially different from the training set. Another consideration is the balance between the number of classes and the number of participants in each class. As demonstrated in our results, inclusion of multiple attributes does not necessarily improve accuracy. This is because the number of classes increases in a factorial way with more attributes, leading to a less accurate predictive power in each class due to small sample sizes. When possible, practitioners should ascertain that, based on some experimental knowledge they might possess, the demographic distribution of the training set would be sufficiently balanced to ensure that a sufficient number of participants would fall in each class. In the absence of an adequate experimental knowledge, a more balanced distribution of the participants in classes can be obtained by coarse-graining the explanatory variables (Garriga, Piera & Bartumeus, 2017). Additionally, the information on the uncertainty associated to the training phase can be propagated to the classification stage toward mitigating the detrimental impact of a small samples size on the accuracy.

It is a common practice in citizen science projects to omit collecting the demographic data of volunteers, and therefore, it is unclear whether the demographics of our participants are comparable to those in typical citizen science. It requires further study to test applicability of our method of using demographics, considering that the demographics are likely to vary depending on the nature of the projects. A further caveat for the application of our method is the necessity of having a gold standard for estimating the conditional probabilities in the training set. This is relevant for applications to binary classification tasks beyond citizen science, as in medical diagnostics, where ground truth is not available (Martinez et al., 2008). In this kind of applications, alternative tools to compare and combine classifiers could be more viable (Keith, Davey & Boyd, 2012).

## Conclusions

This study contributes a solution to the noisy labeler problem, which is common in citizen science. Existing methods require a large sample size to estimate individual reliability (Raykar et al., 2010; Kim & Ghahramani, 2012), which is unfeasible in most citizen science projects with limited effort of volunteers (Nov, Arazy & Anderson, 2011). Our simple, yet effective, algorithm can overcome the problem by focusing on classes of volunteers in a Bayesian framework.

The proposed approach can be readily implemented in citizen science projects by adding a simple survey during the registration to the projects. Although practitioners in citizen science projects may shy away from collecting demographic information from participants in fear of low participation, such information might offer insight into the societal impact of the project by assessing the value of citizen science in education and outreach (Bonney et al., 2009). Similarly, our method can be applied to crowdsourcing for distributed data analysis (Difallah et al., 2015) toward reducing the cost of workers for the same data accuracy, as many crowdsourcing platforms already provide multidimensional, detailed attributes of each worker. Whether it is to gain from limited effort of participants in citizen science or to reduce the cost of crowdsourcing workers, predicting their performance through demographics is a simple, yet powerful, way to improve data accuracy.

## Additional Information and Declarations

### Data Availability

The following information was supplied regarding data availability: Data is available at the Open Science Framework: De Lellis, Pietro. 2019. “Using Demographics toward Efficient Data Classification in Citizen Science: A Bayesian Approach.” OSF. November 3. osf.io/7sqvp.
